# Human Papillomavirus Cervical Infection: Many Ways to a Single Destination

**DOI:** 10.3390/vaccines11010022

**Published:** 2022-12-22

**Authors:** Barbara Gardella, Marianna Francesca Pasquali, Mattia Dominoni

**Affiliations:** 1 Department of Clinical, Surgical, Diagnostic and Paediatric Sciences, University of Pavia, 27100 Pavia, Italy; 2 Department of Obstetrics and Gynecology, IRCCS Fondazione Policlinico San Matteo, 27100 Pavia, Italy

Human papillomavirus (HPV) infection represents the most diffuse sexually transmitted disease of the lower genital tract, with an estimated risk of infection in the general population of 80% over the course of the lifetime [1]. In the pathological pathway of oncogenesis, HPV colonization can result in the development of uterine cervical cancer, vulvar and vaginal cancer, and anal malignancies [2]. Indeed, HPV cervical infection is influenced by the balance between the virus and the host’s immune system: when the virus evades the immune response, it can establish a persistent infection that results in lesion progression and in the production of pro-inflammatory cytokines in the cervico-vaginal microenvironment. In a large percentage of population, HPV infection represents a transitory colonization of cervical tissue, with a spontaneous resolution within 1–2 years (the phenomenon is named viral clearance) without the development of cervical intraepithelial neoplasia (CIN), sustained by appropriate immune response [3,4]. On the other hand, the persistence of high-risk HPV (HR-HPV) infection promotes the development of cervical lesions and high-grade CIN [5].

HPV is able to establish a latent infection, implementing an immunological escape mechanism; after the initial vegetative infection, it is not eradicated by the cell-mediated system and humoral immune response [6]. Moreover, many factors intervene in the interplay between the virus and the host, such as genetics, vaginal microbiota, concomitant lower genital tract infections and host habits (such as smoking). When the virus overcomes host immune response, the infection becomes persistent and develops precancerous and cancerous cellular modifications [6]. Interestingly, HPV is able to synchronize its viral cycle with the differentiation cellular steps of keratinocytes, reducing the immune response and allowing viral proliferation. In addition, circulating immune cells have a reduced activity as long as HPV remains confined to the epithelial tissue, which presents poor vascularization. In this condition, the lack of inflammatory cytokine response reduces the proliferation of Langerhans cells and dendritic cells, with consequent elusion of adaptive immune activation [6]. Concomitantly, HPV silences the expression of cellular factors, such as ND10 factor (sp100), IFT1 (p56), IF16 and APOBEC, which are able to block the viral proliferation and replication [7]. For this reason, knowledge of the interaction between HPV and immune response during viral infection and of the mechanisms underlying the persistence and progression of cervical lesions is essential in order to develop therapeutic strategies that allow adequate protection and prevent the onset of cervical cancer. Literature data suggest that HPV uses different mechanisms to inhibit the innate immune response and pro-apoptotic gene transcription (TRAIL and XAF1). For instance, viral oncoproteins (E6 and E7) suppress different intracellular pathways (e.g., the NfKb pathway), resulting in the decreased secretion and transcription of pro-inflammatory cytokines and chemokines (IL-1, IL-6, tumor necrosis factor–α, Il1β and CCL5) [8,9,10]. The same viral proteins are able to inhibit the transcription of pathogen recognition receptors (PPR) and Toll-like receptors (TLR), abrogating the immunological response of keratinocytes [11,12]. In addition, viral oncoproteins prevent the recruitment of APCs (antigen-presenting cells) such as Langerhans cells, dendritic cells and macrophages, suppressing cytotoxic CD8+ T-cell proliferation and T-cell-mediated immunological activation [13,14,15]. HPV proteins interfere with macrophage translocation and the clearance of viral infected cells through the inhibition of macrophage chemotaxis and monocyte chemotactic protein MCP-1 [16,17]. Eventually, E7 and E5 proteins (sequestering HLA-C and HLA-E antigens) suppress the expression of the MHC1 receptor, reducing cytotoxic cell activity and the consequent elimination of infected tissue by natural killer cells [18,19]. Moreover, E6 and E7 promote the increased production of transforming growth factor β (TGF β), which permits a reduction in local immune response and the progression to oncogenic cell transformation [20]. HPV-related malignancy is sustained by the capacity of HR-HPV to promote CIN progression, the shift from Th1 to Th2 immune response, especially in the early lesions [21], and the increased expression of programmed death-1 PD1 (CD279) molecules and the ligand PD-L1 (CD274), which leads to T-cell apoptosis and functional inability [21,22,23]. Experimental data support the evidence that HPV lesions report elevated levels of regulatory T cells (Treg) expressing the transcriptional factor FOXP3, which confers immune tolerance and auto-immunity preservation, promoting the development of high-grade CIN and progression in the oncogenesis of cervical tissue [24,25].

Recent data confirm that natural HPV infection gives the host a specific immune response (acquired antibodies) to specific genotypes. These antibodies are able to recognize the L1 protein with neutralizing activity and protect against infection, but their production is slow and reported in only a few subjects [26]. For this reason, the main preventive strategy is anti-HPV vaccine, which gives subjects high levels of neutralizing antibodies, and endure for a long time in the serum with an effective humoral response against HPV infection and diseases [27]. This is the key point of the preventive strategy against HPV cervical cancer, but as previous experience has found, new scenarios will appear. In fact, if vaccine administration reduces HR-HPV rate, selective pressure, changes in sexual habits and population geographical migration seem to favor CIN outbreak of negative/untypable or low-grade HPV genotypes [28]. In our opinion, the next-generation screening program should include these less considered categories of HPV types in order to promote the adequate surveillance of cervical cancer prevention.

Nowadays, the possible role of the vaginal microbiome in the evolution of CIN caused by HR-HPV infection represents an interesting approach. Indeed, changes in the normal vaginal microenvironment create a perturbation of local innate immunity and biochemical and structural vaginal and cervical tissue, promoting viral proliferation and reducing clearance [4]. In addition, bacterial vaginosis seems to increase the risk of HPV infection because the modification of the vaginal microbiota alters the vaginal environment [4]. In fact, community state type IV (CST IV) is mostly found in women with bacterial vaginosis; it is characterized by the proliferation of anaerobic bacteria such as *Gardnerella*, *Prevotella* and *Atopobium*, and a reduction in *Lactobacilli.* On the other hand, *Lactobacillus* spp. represent a marker of vaginal health, increasing the production of bacteriocins, biosurfactants, and other protective factors [4]. Moreover, in CIN progression, there is a strong correlation between potential biomarkers, such as Sneathia and Delftia (CST IV and II) and chronic inflammation characterized by pro-inflammatory cytokine overexpression [4]. Analyzing the vaginal metabolome during HPV infection, there is an increment of biogenic amines, glutathione and lipid-related metabolites compared to the absence of viral infection. In addition, in cases of HR-HPV, there is a reduction in the level of amino-acids, lipids and peptides in comparison with low-risk HPV [29].

Improvements in research on the strong connection between HPV infection, the vaginal microenvironment and host immune response, as well as knowledge of viral escape mechanisms, represent the basis of a new approach to reduce HPV-related lesions and promote antiviral strategies able to prevent the infection and transmission of HR-HPV globally.
